# Pharmacokinetics and Dosimetry Studies for Optimization of Pretargeted Radioimmunotherapy in CEA-Expressing Advanced Lung Cancer Patients

**DOI:** 10.3389/fmed.2015.00084

**Published:** 2015-11-27

**Authors:** Caroline Bodet-Milin, Ludovic Ferrer, Aurore Rauscher, Damien Masson, Latifa Rbah-Vidal, Alain Faivre-Chauvet, Evelyne Cerato, Caroline Rousseau, José Hureaux, Olivier Couturier, Pierre-Yves Salaün, David M. Goldenberg, Robert M. Sharkey, Françoise Kraeber-Bodéré, Jacques Barbet

**Affiliations:** ^1^Department of Nuclear Medicine, University Hospital Nantes, Nantes, France; ^2^CNRS UMR 6299, Centre Régional de Recherche en Cancérologie Nantes/Angers (CRCNA), INSERM U892, Nantes, France; ^3^Department of Nuclear Medicine, ICO Cancer Centre, Saint-Herblain, France; ^4^Physics Unit, ICO Cancer Centre, Saint-Herblain, France; ^5^Department of Biochemistry, University Hospital Nantes, Nantes, France; ^6^Department of Pneumology, University Hospital Angers, Angers, France; ^7^Department of Nuclear Medicine, University Hospital Angers, Angers, France; ^8^Department of Nuclear Medicine, University Hospital Brest, Brest, France; ^9^IBC Pharmaceuticals, Inc., Morris Plains, NJ, USA; ^10^Immunomedics, Inc., Morris Plains, NJ, USA; ^11^GIP Arronax, Saint-Herblain, France

**Keywords:** lung cancer, radioimmunotherapy, pretargeting, pharmacokinetics, scintigraphy, SPECT, SPECT/CT, dosimetry

## Abstract

**Objectives:**

A phase I pretargeted radioimmunotherapy trial (EudractCT 200800603096) was designed in patients with metastatic lung cancer expressing carcinoembryonic antigen (CEA) to optimize bispecific antibody and labeled peptide doses, as well as the delay between their injections.

**Methods:**

Three cohorts of three patients received the anti-CEA × anti-histamine-succinyl-glycine (HSG)-humanized trivalent bispecific antibody (TF2) and the IMP288 bivalent HSG peptide. Patients underwent a pretherapeutic imaging session S1 (44 or 88 nmol/m^2^ of TF2 followed by 4.4 nmol/m^2^, 185 MBq, of ^111^In-labeled IMP288) and, 1–2 weeks later, a therapy session S2 (240 or 480 nmol/m^2^ of TF2 followed by 24 nmol/m^2^, 1.1 GBq/m^2^, of ^177^Lu-labeled IMP288). The pretargeting delay was 24 or 48 h. The dose schedule was defined based on preclinical TF2 pharmacokinetic (PK) studies, on our previous clinical data using the previous anti-CEA-pretargeting system, and on clinical results observed in the first patients injected using the same system in Netherlands.

**Results:**

TF2 PK was represented by a two-compartment model in which the central compartment volume (Vc) was linearly dependent on the patient’s surface area. PK was remarkably similar, with a clearance of 0.33 ± 0.03 L/h/m^2^. ^111^In- and ^177^Lu-IMP288 PK was also well represented by a two-compartment model. IMP288 PK was faster (clearance 1.4–3.3 L/h). The Vc was proportional to body surface area, and IMP288 clearance depended on the molar ratio of injected IMP288 to circulating TF2 at the time of IMP288 injection. Modeling of image quantification confirmed the dependence of IMP288 kinetics on circulating TF2, but tumor activity PK was variable. Organ-absorbed doses were not significantly different in the three cohorts, but the tumor dose was significantly higher with the higher molar doses of TF2 (*p* < 0.002). S1 imaging predicted absorbed doses calculated in S2.

**Conclusion:**

The best dosing parameters corresponded to the shorter pretargeting delay and to the highest TF2 molar doses. S1 imaging session accurately predicted PK as well as absorbed doses of S2, thus potentially allowing for patient selection and dose optimization.

**Trial Registration:**

ClinicalTrials.gov NCT01221675 (EudractCT 200800603096).

## Introduction

Radioimmunotherapy (RAIT) is a molecular targeted therapy whereby irradiation from radionuclides is delivered to target tumors using monoclonal antibodies (mAb) directed to tumor antigens. RIT delivers a heterogeneous low dose-rate irradiation with an efficacy demonstrated in hematological malignancies sensitive to radiation therapy (1). In solid tumors, more resistant to radiation and less accessible to large molecules, such as mAb, clinical efficacy remains limited and fractionated injections, combination of RAIT with chemotherapy, as well as pretargeting approaches, are being studied to improve antitumor efficacy (2).

Pretargeted RAIT (pRAIT) was originally designed to improve the therapeutic index (tumor-to-normal tissue ratios) and to deliver increased absorbed doses to tumors, as compared to directly radiolabeled antibodies or antibody fragments (3, 4). pRAIT may be achieved in several different ways. Here, a bispecific mAb (BsmAb) is administered, followed a few days later by a radiolabeled bivalent hapten. With this technology, the radioactive bivalent hapten binds avidly to the BsmAb attached to the cell surface, whereas the nontargeted radioactive hapten clears from the circulation through the kidneys. RAIT using directly radiolabeled anti-carcinoembryonic antigen (CEA) mAb has shown promising clinical results in metastatic medullary thyroid carcinoma (MTC) and metastatic colon cancer (5, 6), but pretargeting of CEA-expressing tumors has demonstrated a more favorable therapeutic index and antitumor efficacy in preclinical MTC and colorectal cancer (CRC) models (7, 8) and clinical feasibility in MTC and small-cell lung cancer (SCLC) (9, 10). Two phase I clinical trials assessing anti-CEA × anti-diethylene-triamine-pentaacetic acid (DTPA)-indium BsmAb (murine F6 × 734 and chimeric hMN14 × 734 BsmAb) with ^131^I-labeled di-DTPA-indium hapten showed encouraging therapeutic results in patients with progressive metastatic MTC, with a significantly improved overall survival for intermediate- and high-risk patients (11). However, murine and chimeric BsmAb (human/mouse) used in these studies induced in a high rate of immunization and 26% human antihuman antibody (HAHA) or human antimouse antibody (HAMA) detection, as reported by Salaün et al. (12).

New generation humanized, recombinant, trivalent BsmAb (anti-CEA TF2) and histamine-succinyl-glycine (HSG) peptides have thus been developed (13). TF2, composed of a humanized anti-HSG Fab fragment derived from the 679 anti-HSG mAb and two humanized anti-CEA Fab fragments derived from the hMN14 mAb (labetuzumab; Immunomedics, Inc.) by the dock-and-lock procedure, should reduce immunogenicity and facilitate repeated injections (14, 15). Moreover, the HSG peptide, IMP288, allows facile and stable labeling with different radiometals, such as ^177^Lu and ^90^Y, having favorable physical features that could improve pRAIT efficacy. However, Schoffelen et al. (16) have shown that doses and pretargeting delays must be entirely revisited with the dock-and-lock BsmAb because of the very different pharmacokinetic (PK) properties of these new agents, as compared to the chemically coupled Fab fragments used previously (17).

A phase I/II clinical trial was designed to optimize and assess, in CEA-expressing lung cancer patients, the new generation pretargeting reagents, i.e., the anti-CEA × anti-HSG TF2 BsmAb, and the radiolabeled IMP288 HSG peptide. The clinical protocol includes two parts: the first part aims at optimizing BsmAb and peptide molar doses and administration schedules for pRAIT in three cohorts of three patients, using detailed PK and dosimetry analyses, and the second part aims at determining the maximum tolerated dose of pRAIT using escalated peptide activities and the parameters optimized in the first study part. In the two parts of the study, the IMP288 peptide is radiolabeled with indium-111 for pretherapeutic imaging and lutetium-177 for therapy. Here, we report the results of the optimization part of the study, performed in three cohorts of patients receiving different doses of BsmAb and peptide, the ratio of BsmAb and peptide molar doses being kept constant between the imaging and therapy sessions, as suggested by Schoffelen et al. (16). A population PK approach was used to model the serum kinetics of the BsmAb and of the radiolabeled hapten for the two sessions. Whole body (WB) planar scintigraphy and single photon emission computed tomography (SPECT) allowed the description of the biodistribution of the radiolabeled peptide and quantitative imaging analyses. Dosimetry assessment was performed together with population PK analysis of the time–activity curves.

## Materials and Methods

### Patients

The target population was male or female 18 years of age with histological diagnosis of CEA-positive lung cancer including

- small-cell lung cancer who are in partial response or who failed after at least two lines of standard radiation and/or chemotherapy;
- non-small-cell lung cancer (NSCLC) without activating mutation of epidermal growth factor receptor (EGFR) gene and who failed after at least one line of chemotherapy.

Only patients with CEA serum level ≥10 ng/mL or CEA expression by tissues staining, with at least one known tumor site detected by computed tomography (CT) and positron emission CT using ^18^F-fluoro-deoxy-glucose (FDG-PET), were eligible for the study. All patients had an Eastern Cooperative Oncology Group performance ≤2 or Karnofsky performance status ≥60% and a minimum life expectancy of 3 months. For entry into the study, patients were required to be at least 4 weeks beyond any major surgery, external radiotherapy, chemotherapy, immunotherapy, or angiogenesis inhibitor therapy. The patients were required to have normal levels of transaminases (AST and ALT ≤ 2.5 × the upper limit of normal), total bilirubin level ≤30 mmol/L, creatinine (≤2.5 × the upper limit of normal), neutrophils ≥1,500/mL, and platelets ≥100,000/mL. Pregnant or breast-feeding women were excluded, as were premenopausal women not willing to practice adequate birth control methods during the study and for 3 months afterward. Patients with another known type of intercurrent cancer, uncontrolled diabetes, or a psychiatric disorder were also excluded.

All patients gave informed written consent in accordance with institutional guidelines, including the Declaration of Helsinki. The trial was approved by the responsible ethics committee and registered at ClinicalTrial.gov NCT01221675 (EudractCT 200800603096).

### Investigational Products and Labeling

The trivalent TF2-humanized mAb and the IMP288 peptide, which bears two HSG groups (hapten) recognized by the 679 mAb and one 1,4,7,10-tetraazacyclododecane-1,4,7,10-tetraacetic acid (DOTA) moiety (13, 18), were prepared suitable for human use by Immunomedics, Inc. (Morris Plains, NJ, USA). IMP288 (146 μg/mL in acetate buffer) was labeled with 185 MBq of ^111^In (Mallinckrodt Medical B.V., Petten, Netherlands) for the imaging sessions. IMP288 (24 mol/m^2^) was labeled with 1.1 GBq/m^2^ of ^177^Lu (IDB Radiopharmacy B.V., Baarle-Nassau, Netherlands) for the therapy sessions. The radiochemical purity, determined using high performance liquid chromatography (Eckert Ziegler, Germany) using a C18 column (ACE 15 cm × 3 mm, France) and a gradient of trifluoroacetic acid (0.1% in water) and acetonitrile was greater than 90% (94.5 ± 2.2%) for ^111^In-IMP288 and greater than 99.0% (99.5 ± 0.4%) for ^177^Lu-IMP288. TF2 was diluted in 250 mL 0.9% NaCl and administered by i.v. infusion over a period of 30–60 min. ^111^In- or ^177^Lu-IMP288 was diluted in 50 mL of 0.9% NaCl in water and administered by i.v. infusion over a period of 30 min. Median-specific activities were 24 MBq/nmol (range 16–30) for ^111^In-IMP288 and 47 (range 45–53) for ^177^Lu-IMP288.

### Study Design and Treatment

Three different pretargeting conditions were examined in three cohorts of three patients (Table 1). All patients underwent a pretherapy imaging session (S1) using TF2 and ^111^In-labeled IMP288 injections before a therapy session (S2). In the first cohort (C1), patients received 7 mg/m^2^ (44 nmol/m^2^) of TF2 followed 48 h later by 4.4 nmol/m^2^ of IMP288 labeled with 185 MBq of ^111^In in S1 and 37.5 mg/m^2^ (240 nmol/m^2^) of TF2 followed 48 h later by 24 nmol/m^2^ of IMP288 labeled with 1.1 GBq/M2 of ^177^Lu in S2. Only patients with successful tumor targeting in S1 were eligible to participate in S2. In the second cohort (C2), TF2 doses were increased from 7 (44 nmol/m^2^) to 14 mg/m^2^ (88 nmol/m^2^) in S1 and from 37.5 (240 nmol/m^2^) to 75 mg/m^2^ (480 nmol/m^2^) in S2, whereas IMP doses, ^111^In and ^177^Lu activities, and the pretargeting delay remained identical that in C1. In the third cohort (C3), patients received the same TF2 and IMP doses and the same ^111^In and ^177^Lu activities than in C2 but with a lower pretargeting interval (24H instead of 48H). For each cohort of patients, TF2 and IMP288 were administered using the same pretargeting interval and the sameTF2/IMP288 molar ratio in S1 and S2.

**Table 1:** **Dosing scheme**.

	S1: pretherapy imaging session			S2: therapy session		
	TF2 dose	Delay (h)	^111^In-IMP288	TF2 dose	Delay (h)	^177^Lu-IMP288
Cohort I	7 mg/m^2^	48	185 MBq	37.5 mg/m^2^	48	1.1 GBq/m^2^
	44 nmol/m^2^		4.4 nmol/m^2^	240 nmol/m^2^		24 nmol/m^2^
Cohort II	14 mg/m^2^	48	185 MBq	75 mg/m^2^	48	1.1 GBq/m^2^
	88 nmol/m^2^		4.4 nmol/m^2^	480 nmol/m^2^		24 nmol/m^2^
Cohort III	14 mg/m^2^	24	185 MBq	75 mg/m^2^	24	1.1 GBq/m^2^
	88 nmol/m^2^		4.4 nmol/m^2^	480 nmol/m^2^		24 nmol/m^2^

Six French centers were allowed to include and treat patients in this multicentric study: Nantes University Hospital Nuclear Medicine Department, Nantes ICO Cancer Centre Nuclear Medicine Department, Brest University Hospital Nuclear Medicine Department, Angers University Hospital Nuclear Medicine Department, Clermont-Ferrand University Hospital Nuclear Medicine department, and Grenoble University Hospital Nuclear Medicine Department.

Safety was assessed during infusions by monitoring vital signs, physical examination, and adverse events. Patients were premedicated with antihistamine (xyzall^®^) and corticosteroid (intravenous dexamethasone) before each TF2 and peptide infusion.

NCI Common Toxicity Criteria Version 3.0 were used to evaluate toxicity. Total WBC and lymphocytes were monitored and reported every week until 8 weeks post-^177^Lu-IMP288 injection or until platelet (>100 g/L without transfusion), hemoglobin (>10 g/dl without transfusion), and leukocyte (>2 g/L) recovery. Assessment of hematological toxicity was only based on hemoglobin level (Hgb), absolute neutrophil counts, and platelet counts. Biochemical tests including serum creatinine, creatinine clearance, AST, ALT, total bilirubin, alkaline phosphatase, calcium, phosphorus, uric acid, sodium, potassium, and serum electrophoresis were performed 4 weeks, 8 weeks, and then every 3 months after pRAIT.

Assessment of response was based on physical examination, CEA serum level, CT and FDG-PET performed 4 weeks after pRAIT, at 3 months, and then every 3 months until progression. Responses were scored according to the Response Evaluation Criteria in Solid Tumors (RECIST 1.0) (19).

Human antihuman antibody was determined within 2 days of the second TF2 infusion, then 4 weeks, 8 weeks, and 3 months after the last TF2 infusion using an ELISA method. The detection limit for positive HAHA was 50 ng/mL.

### Pharmacokinetics

Blood samples were collected in separator tubes for serum collection at the following times after TF2 injection and after administration of ^111^In-IMP288 or ^177^Lu-IMP288: before the beginning of the infusion, 5 min before the end of the infusion, then 5 min, 1 h, 2–4 h, 24 h, and then at four other times over 7 days. Blood samples were collected for all patients during S1 and S2. Serum samples (at least 1 mL) were prepared from blood samples and stored frozen. TF2 concentrations were determined using an ELISA (Immunomedics), as described previously (18). The indium-111 or lutetium-177 activity in each serum sample was determined by counting 0.1–0.2 mL of serum in a calibrated gamma counter. Counting was performed immediately after the end of each blood collection series and corrected for radioactive decay.

Modeling of the serum concentration PK was performed using a two-compartment model for the bispecific antibody (TF2) and two or three compartment models for the radiolabeled hapten (IMP288), using a population PK software package, developed in the laboratory and validated against Monolix (20). Patients’ body surface areas (BSAs) were used as covariables. In the hapten PK analyses, several covariables were tested to represent the effect of TF2 on the kinetics of IMP288. Finally, the molar ratio of injected hapten to the amount of TF2 present in the patients’ serum (MR), calculated as the concentration of TF2 extrapolated to the time of hapten injection multiplied by the central compartment volume (Vc) obtained in the TF2 PK analysis, was the covariable used to take the effect of TF2 on IMP288 PK into account. Since the addition of a third compartment did not improve data fitting, two-compartment models were used. Parameters for the two-compartment model of serum kinetics for both TF2 and IMP288 were the transfer rates (*k*_2,1_ and *k*_1,2_), the elimination rate (*k*_el_), and the Vc per BSA unit (volume/m^2^). The Vc was a dependent parameter (Vc = volume/m^2^ × patient BSA). For the serum IMP288 PK, clearance was calculated as A_S_ × MR^BS^, MR being the ratio of the number of moles of injected IMP288 to the number of moles of TF2 present in the circulation at the time of IMP288 injection, *A*_S_ and *B*_S_ being two adjustable parameters, and *k*_el_ was calculated as clearance/Vc.

For the WB IMP288 kinetics, the activity calculated from WB images was modeled as the sum of the central and distribution compartments with adjustable transfer rates (*k*_2,1_ and *k*_1,2_) and two additional adjustable parameters, *A*_WB_ ad *B*_WB_, used to calculate the elimination rate: $k_{\mathrm{el}}=A_{\mathrm{WB}}\times {\mathrm{MR}}^{B_{\mathrm{WB}}}$.

For the PK analysis of tissue distribution from image quantification, the WB kinetics was used as an input function, and the IMP288 distribution in tissues of interest was modeled using a tissue-specific distribution compartment and a fraction of the activity in the central compartment. The on and off rate constants, *k*_on_ and *k*_off_, and the fraction of activity (fraction) were adjustable parameters.

### Scintigraphy

All imaging centers were equipped with the same model of SPECT/CT gamma-camera (Symbia T/T2, Siemens). For pretherapeutic and therapeutic imaging sessions, three to five images were scheduled from 1 h to 7 days after radiopharmaceutic injection depending on the patient’s ability to sustain imaging procedures. Each imaging session consisted of WB emission scan (256 × 1,024 pixels) and tomographic acquisitions (128 × 128 pixels, 2 × 32 projections, and 30–45 s per projection). Each scans or tomographic acquisitions were performed using medium energy collimators. Due to expected low level of detected counts, especially at late time points, energy windows were centered on the two major peaks (15% width). In order to correct for Compton scattering (21), two energy windows (4% width) adjacent to each main peak were simultaneously acquired. CT scans (120 kVp, 100 mAs) were acquired in order to derive CT attenuation maps. Tomographic procedures consisted of two acquisitions to cover patients from lungs to pelvic area.

At an initial stage, as involved centers were equipped with thin (3/8″) or thick (5/8″) crystals, system planar sensitivity was evaluated for these two types of equipment for ^111^In and ^177^Lu sources of known activity (~200 and 900 MBq, respectively) poured in a thin cylinder plate (Table 2). As tomographic reconstructions accounted for point response function of the collimator, this feature was characterized by the full width of half-maximum of point source images at different distances from collimator plate. Gamma cameras and dose calibrator quality controls were performed in accordance to each institution procedures (no crosscalibration). Particular attention was given to patient alignment for each imaging session to help image registration during processing.

**Table 2:** **^111^In and ^177^Lu system sensitivities for both types of gamma cameras in all centers**.

System sensitivity (counts/MBq s)		
Crystal thickness	^111^In	^177^Lu
3/8″	170 ± 2	15 ± 2
5/8″	230 ± 2	18 ± 2

### Dosimetry

Quantitative imaging was performed on tomographic reconstructions (OSEM 30 iterations, eight subsets) taking into account attenuation, Compton scattering (correction performed on projections), and collimator response corrections as expressed in international guidelines (22). Initial estimated system sensitivity reported in Table 2 was used to translate detected count into activity for ^111^In and ^177^Lu acquisitions.

Patients’ organ masses were derived from automatic or manual CT images segmentation performed with 3D Slicer (23) software. Trabecular bones in L2–L4 lumbar vertebras were also segmented in order to evaluate bone marrow-absorbed dose (24). Tumor volumes were segmented on emission-reconstructed images and performed on images where the tumor-to-background ratio was visually designed as optimum. Tumor labeled volumes were subsequently exported as DICOM-RT structure set and integrated into the patient labeled-organs images after registration.

In order to obtain time–activity curves for volumes of interest, labeled images were registered against CT images performed at every imaging session using the MedInria software (25). Then, organ time–activity curves were adjusted with nls package of R software (26) using mono- or biexponential functions depending on the number of time points available to perform the regression analysis and submitted to a population PK analysis using the laboratory software package.

To estimate patient’s organ-absorbed doses at pretherapeutic and therapeutic stages, MIRD S factors were scaled by patient organ masses. Time integration of fitted functions was calculated to derive cumulated activities at both sessions. Estimations at pretherapeutic were scaled to take into account the difference between physical half-lives of both radionuclides.

### Statistics

Organ effective periods estimated at S1 and S2, as well as tumor- and organ-absorbed doses for each schedule, were compared using the Wilcoxon statistical test.

For each patient, Spearman statistical tests were conducted to evaluate whether S1-absorbed doses were able to predict absorbed doses during S2.

## Results

### Patient Characteristics and Therapy Results

Ten patients were included in the study between June 2011 and September 2014 (one patient included in Brest, two patients in Angers, two patients in Nantes University Hospital, and five patients in Nantes ICO Cancer Centre) and nine were treated; one on the five patients included in Nantes ICO Cancer Centre died between the inclusion and the beginning of the study. Characteristics of the nine treated patients are summarized in Table 3. All patients received 185 MBq of ^111^In-IMP288 for the pretherapeutic session S1 and a median activity of 2,147 MBq (1,641–3,026 MBq) of ^177^Lu-IMP288 for the therapeutic session S2, one or 2 weeks later. None of the nine patients experienced an anaphylactic reaction during TF2 or peptide infusion. One patient died 5 days after pRAIT (not considered treatment related) and was not evaluable for PK analysis, dosimetry, toxicity, or response assessment.

**Table 3:** **Characteristics of patients**.

Median age	65 (53–80)
Male/female	7/2
Histological type	6 SCLC/3 NSCLC
Karnofsky index	
90–100	5
70–80	2
60–70	2
Median CEA plasma level (min–max)	79 ng/mL (10–388)
Prior treatment	
Chemotherapy	100%
Radiotherapy	67%
Surgery	33%
Tyrosine kinase inhibitors	11%
Site of disease	
Lung	78%
Mediastinum	78%
Liver	56%
Pancreas	22%
Adrenal	33%
Infradiaphragmatic nodes	33%
Bone	11%
Muscle	11%
Brain	11%
Median delay from initial diagnosis (min–max)	25 months (10–64)

Bone marrow toxicity was mild in most patients (grade 1 thrombocytopenia in 2/9 patients). Only one patient experienced grade 2 anemia 3 months after ^177^Lu administration. Three patients with liver metastases showed transaminase enzymes (AST and ALT) elevation during the follow-up, which was deemed to be disease-related, since the three patients had progression of liver metastases on CT and FDG-PET registered 4 weeks after pRAIT. None of the patients showed biological signs or symptoms of renal toxicity.

According to RECIST criteria based on FDG-PET and CT data, two patients were considered as stable at 4 weeks post-pRAIT but progressive at 3 months. The six others patients were progressive as soon as 4 weeks after pRAIT.

Human antihuman antibody elevation was detected in 1/8 patients, 1 month after the second TF2 infusion, gradually decreasing from 2,966 ng/mL at 4 weeks to 969 ng/mL in the follow-up period of 3 months.

### TF2 Pharmacokinetics

**Figure 1:**
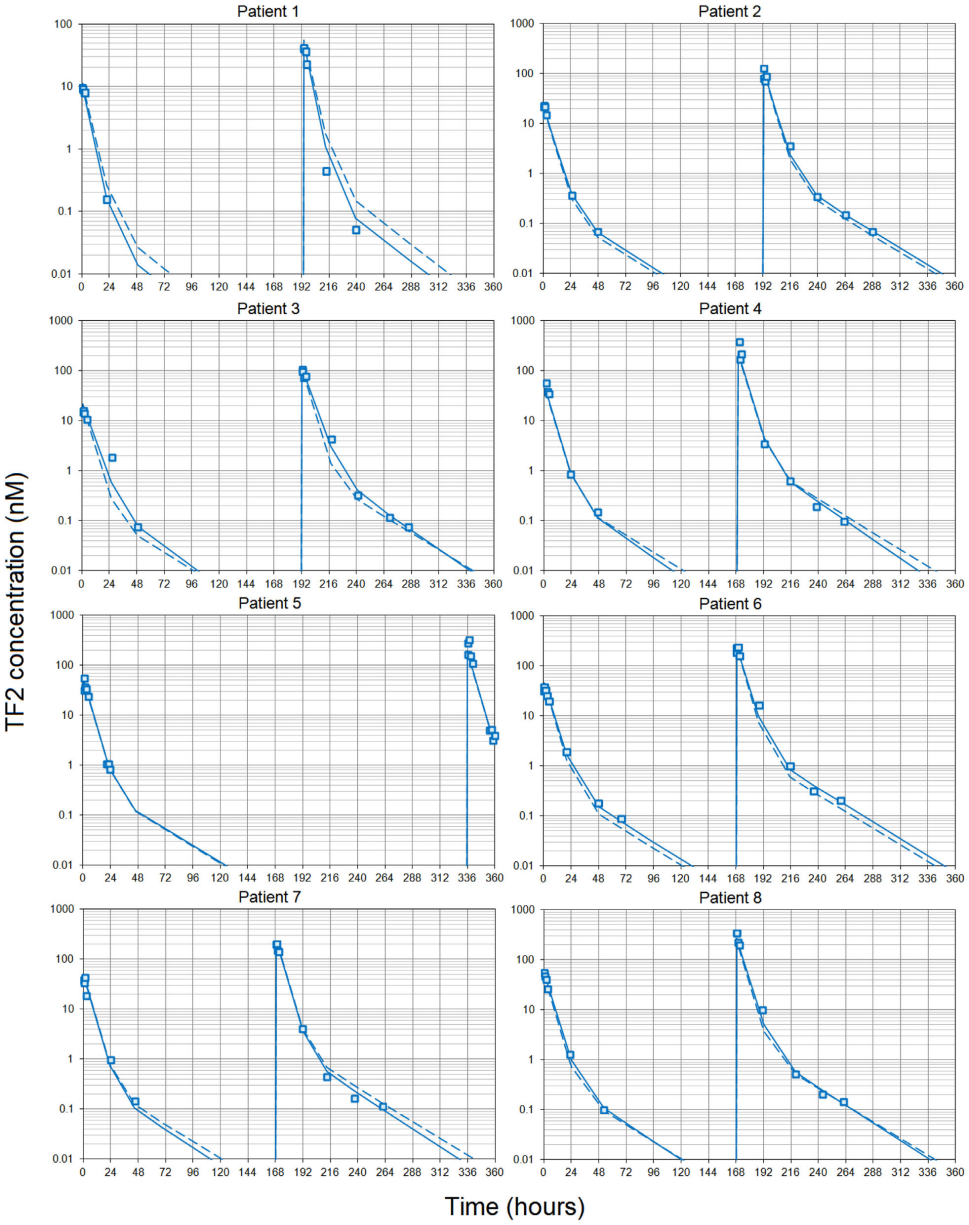
**Pharmacokinetics of the bispecific antibody TF2**. Each patient received two infusions of TF2 at 7 or 8 days intervals (except patient 5). Blood samples were collected at selected time intervals during and after each infusion and centrifuges. TF2 concentrations were measured using a specific ELISA. The pharmacokinetics was then modeled using a two-compartment model and a population approach. Data collected after both infusions were fitted using a single set of parameters. Results (open squares) are plotted as a semilog plot with the population (dashed lines) and individual (solid lines) fitted curves.

Simultaneous modeling of the two TF2 infusions for the eight patients who completed both S1 and S2 sessions using a two-compartment model showed that the PK was consistent between the two infusions, even if they were given at different TF2 molar doses (Figure 1). The use of population PK and the simultaneous modeling of the S1 and S2 TF2 infusions surmounted the problem of the limited sensitivity of the ELISA (Table 4). The small differences observed between the population and the individual fits showed that interindividual variability was quite small. The serum kinetics was rather fast with mean alpha half-lives of 3.7 ± 0.1 h and beta half-lives of 21.3 ± 0.7 h, with very low interindividual variability (3.2 and 3.3%, respectively). Mean serum clearance was 0.64 ± 0.12 L/h, and the use of BSA as a covariable (by setting Vc = VBA × BSA) reduced the coefficient of variation of the Vc from 19 to 4.0% for the estimated parameter (VBA). This observation validates *a posteriori* the dosing scheme of the dose escalation on a BSA basis (44/88 nmol/m^2^ for S1 and 240/480 nmol/m^2^ for S2).

**Table 4:** **Two-compartment population analysis of TF2 pharmacokinetics**.

Parameter^a^	*k*_2,1_ (h^−1^)	*k*_1,2_ (h^−1^)	*k*_el_ (h^−1^)	Volume/m^2^ (L/m^2^)	Vc (L)	Clearance (L/h)
**Population values**						
Estimation	0.034	0.0075	0.182	1.86	NA	NA
SD	0.002	0.0005	0.003	0.04	NA	NA
**Individual values**						
Patient 1	0.033	0.0062	0.194	2.00	4.01	0.78
Patient 2	0.033	0.0080	0.177	1.82	2.71	0.48
Patient 3	0.036	0.0081	0.169	1.83	4.93	0.84
Patient 4	0.036	0.0071	0.182	1.80	3.33	0.61
Patient 5	0.034	0.0075	0.1.83	1.81	3.05	0.56
Patient 6	0.033	0.0085	0.174	1.84	3.20	0.56
Patient 7	0.035	0.0068	0.183	1.92	3.73	0.68
Patient 8	0.035	0.0073	0.177	1.77	3.45	0.61
Mean^b^	0.034	0.074	0.180	1.85	3.52	0.64
SD	0.001	0.001	0.007	0.07	0.69	0.12
CV (%)	5.5	14.5	9.0	7.7	21.4	20.1

*^a^The transfer rates (*k*_2,1_ and *k*_1,2_), the elimination rate (*k*_el_), and the central compartment volume per body surface area unit (m^2^) were adjusted to a two-compartment model. The central compartment volume was a dependent parameter (Vc = volume/m^2^ × patient body surface area) as well as clearance (clearance = Vc × *k*_el_)*.

*^b^The mean, SD, and CV for each parameter were calculated from individual estimations. Note the low interindividual variability and the lower CV of the adjusted volume per square meter compared to that of the actual central compartment volumes (Vc)*.

### IMP288 Pharmacokinetics

Modeling the kinetics of the hapten was complicated by the necessity to take into account the effect of the residual bispecific antibody in serum at the time of hapten administration, which binds the hapten and modulates its clearance. To compare the PK of IMP288 labeled with indium-111 and with lutetium-177, indium activities were corrected for radioactive decay and transformed into equivalent lutetium-177 counts, assuming similar PK for IMP288 labeled with the two radionuclides (16). Then, the time–activity curves were fitted individually for all patients to a two-compartment model, which gave a good visual fit, not significantly improved by a third compartment according to the Akaike criterion (not shown). In a second step, the relationship between IMP288 PK and the pretargeting conditions was tested by plotting the estimated clearance or the Vc against the concentration of TF2 at the time of IMP288 injection (interpolated from the fitted TF2 concentration curves), or the amount of TF2 present in the circulation at the time of IMP288 (calculated as TF2 concentration × TF2 Vc), or the molar ratio of injected IMP288 to the amount of TF2 in the circulation (MR). Indeed, in the circulation, TF2 binds the IMP288 hapten and slows its clearance. It seems logical that the lower the excess of IMP288 relative to TF2, the larger the trapping of IMP288 in the circulation by the bispecific antibody, and hence, the slower its clearance. The correlation based on a power relationship was found to be better between clearance and MR, which was used thereafter as a covariable in the population analysis.

**Figure 2:**
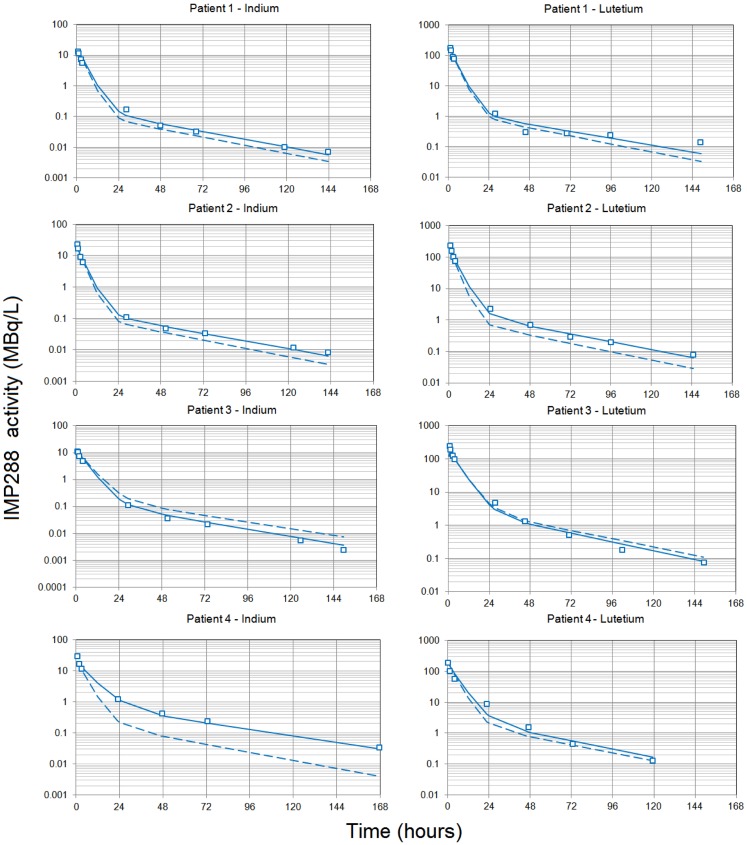

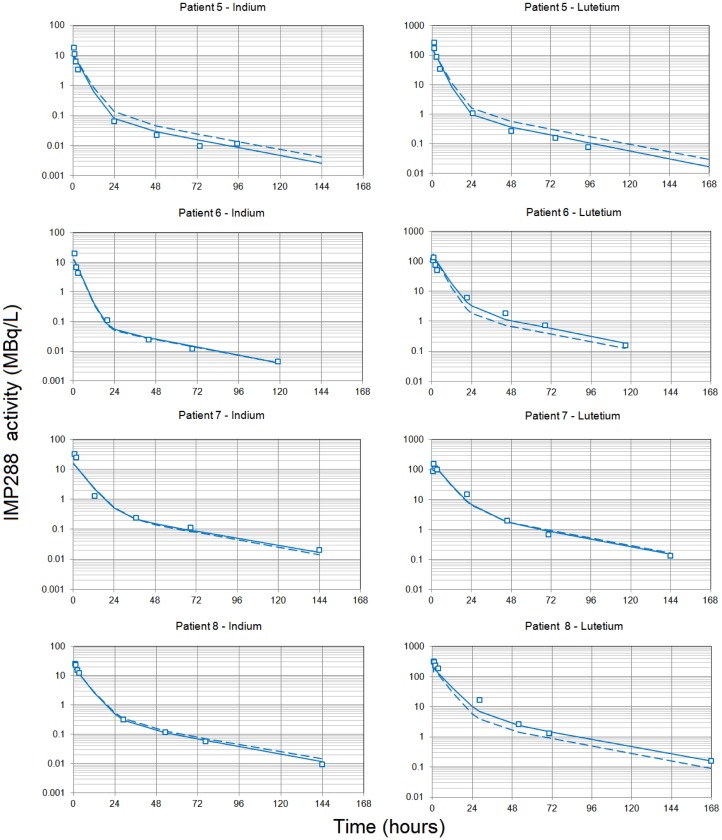
**Pharmacokinetics of the labeled hapten IMP288**. Each patient received TF2 infusions then 24 or 48 h after each infusion, they received an infusion of IMP288 labeled with indium-111 after the first TF2 infusion or labeled with lutetium-177 after the second. Blood samples were collected at selected time intervals during and after each infusion, centrifuged, and counted. Indium-111 counts were corrected to match lutetium-177 radioactive half-life, and the figure shows IMP288 activity concentrations. The pharmacokinetics was modeled using a two-compartment model and a population approach. Results (open squares) are plotted as a semilog plot with the population (dashed lines) and individual (solid lines) fitted curves.

A population PK analysis was then performed on all 16 available kinetics, using BSA and MR as covariables. The larger interindividual variability in the IMP288 than in TF2 kinetics, with mean alpha half-lives of 3.4 ± 0.8 h and beta half-lives of 28.9 ± 2.1 h (corresponding to CV of 24 and 7.3%, respectively), could be explained in part by the influence of TF2 predose. The IMP288-indium-111 kinetics for patient 4 appeared as an outlier (Figure 2) but was not excluded from the analysis. The PK of the hapten is known to depend on the presence of TF2 in body fluids, and a strong correlation had been described earlier between IMP288 blood residence time and the concentration of TF2 blood concentrations at the time of peptide injection (16). Since the individual fitting analysis pointed to a relationship between hapten clearance and MR, MR was introduced in the population analysis as a covariable, and IMP288 clearance was calculated as $\mathrm{CI}=A_{B}\times {\mathrm{MR}}^{B_{B}}$ and *k*_el_ as clearance/Vc (Table 5). Parameter adjustment finally gave clearance (L/h) = 1.33 × MR^0.18^ (*R*^2^ = 0.66). As expected, IMP288 clearance was higher than that of TF2, varying from 1.36 to 3.25 L/h, depending on MR, thus on the pretargeting conditions.

**Table 5:** **Two-compartment population analysis of IMP288 pharmacokinetics**.

Parameter^a^	*k*_2,1_ (h^−1^)	*k*_1,2_ (h^−1^)	*A*_S_	*B*_S_	MR	*k*_el_ (h^−1^)	Volume/m^2^ (L/m^2^)	Vc (L)	Clearance (L/h)
**Population values**									
Estimation	0.027	0.019	1.42	0.182	NA	NA	6.38	NA	NA
SD	0.001	0.002	0.07	0.012	NA	NA	0.27	NA	NA
**Individual values**									
Patient 1, indium	0.027	0.021	1.35	0.169	124.2	0.226	6.69	13.4	3.04
Patient 1, lutetium	0.024	0.021	1.37	0.172	125.5	0.241	6.50	13.1	3.15
Patient 2, indium	0.026	0.023	1.31	0.167	40.7	0.250	6.54	9.7	2.43
Patient 2, lutetium	0.027	0.022	1.22	0.156	40.1	0.219	6.63	9.9	2.16
Patient 3, indium	0.028	0.015	1.53	0.199	35.7	0.185	6.23	16.8	3.11
Patient 3, lutetium	0.028	0.017	1.43	0.183	37.8	0.165	6.25	16.8	2.78
Patient 4, indium	0.026	0.026	0.92	0.141	22.6	0.112	6.90	12.7	1.43
Patient 4, lutetium	0.029	0.019	1.29	0.167	22.0	0.173	6.78	12.5	2.17
Patient 5, indium	0.027	0.017	1.53	0.196	22.5	0.235	6.15	12.0	2.82
Patient 5, lutetium	0.028	0.017	1.52	0.196	31.1	0.249	6.13	11.9	2.97
Patient 6, indium	0.027	0.019	1.41	0.179	109.0	0.291	6.44	11.2	3.25
Patient 6, lutetium	0.028	0.021	1.31	0.170	18.8	0.179	6.93	12.0	2.15
Patient 7, indium	0.027	0.022	1.41	0.181	3.3	0.146	6.21	12.0	1.76
Patient 7, lutetium	0.028	0.018	1.41	0.181	3.2	0.137	6.54	12.7	1.74
Patient 8, indium	0.028	0.018	1.44	0.182	2.3	0.161	6.18	10.4	1.68
Patient 8, lutetium	0.028	0.020	1.16	0.174	2.5	0.124	6.49	10.9	1.36
Mean^b^	0.027	0.020	1.35	0.176		0.193	6.47	12.4	2.37
SD	0.001	0.003	0.16	0.015		0.052	0.27	2.0	0.65
CV (%)	4.5	14.3	11.5	8.6		27.0	4.1	16.4	27.5

*^a^The transfer rates (*k*_2,1_ and *k*_1,2_), the central compartment volume per body surface area unit (m^2^), and the two parameters *A* and *B* were adjusted using a two-compartment model. Estimations of the population adjusted parameters are given together with their estimated SD. The central compartment volume was a dependent parameter (Vc = volume/m^2^ × patient body surface area). Clearance was calculated as *A*_S_ × MR^BS^, MR being the ratio of the number of moles of injected IMP288 to the number of moles of TF2 present in the circulation at the time of IMP288 injection and the elimination constant *k*_el_ as clearance/Vc*.

*^b^The mean, SD, and CV for each parameters were calculated from individual estimations*.

Since IMP288 kinetics were primarily driven by MR, and given the low interindividual variability of TF2 kinetics, pretargeting parameters (i.e., molar injected doses of TF2 and IMP288 and pretargeting delay) are expected to control the hapten kinetics. This means that the kinetics of the therapeutic session may be predicted by the imaging session, in spite of the fact that absolute molar doses of the reagents are different, which will be confirmed hereafter.

### Scintigraphy and Quantitative Analyses

**Figure 3:**
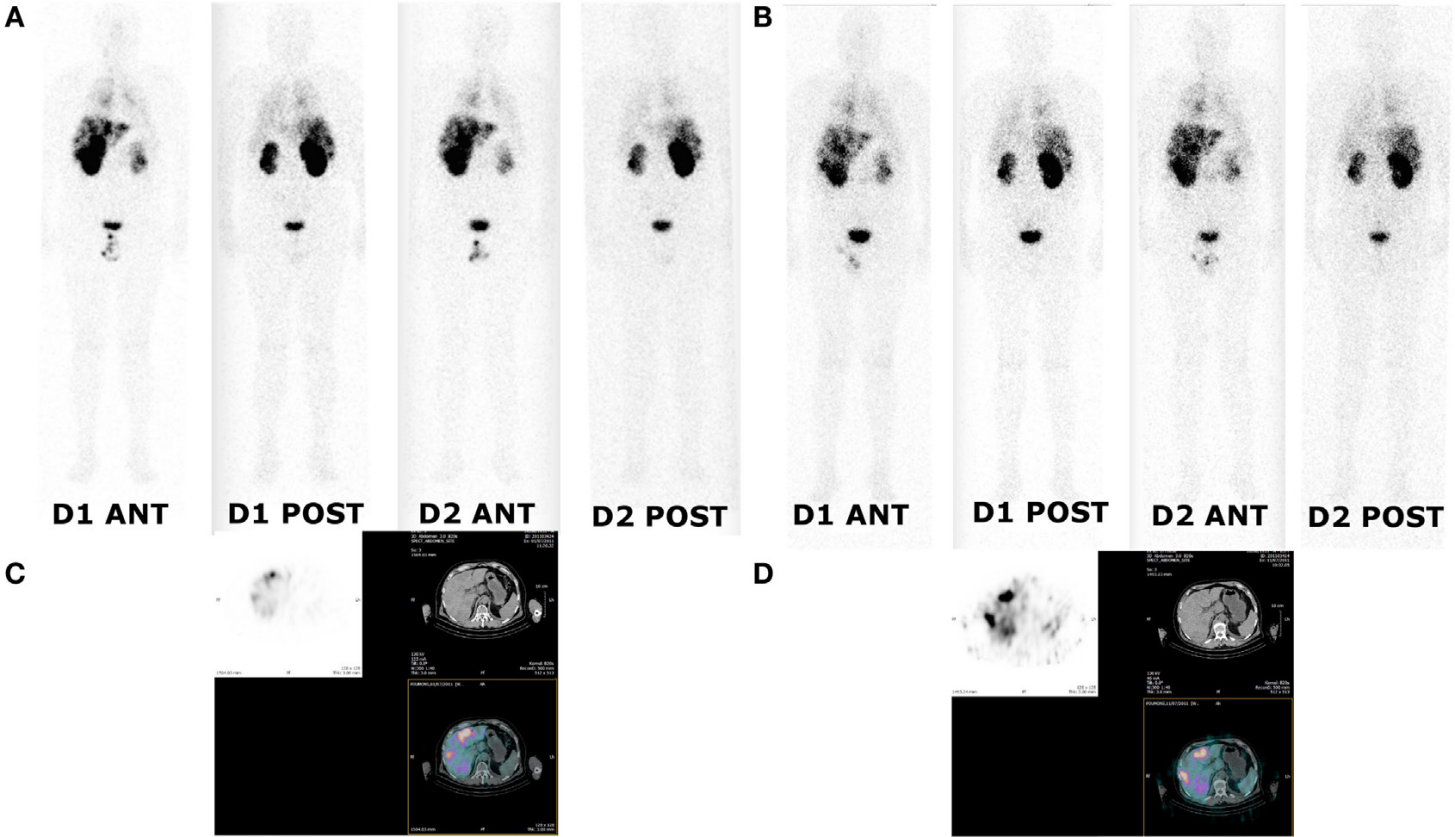
**Whole body and SPECT/CT images of patient 1 (cohort I)**. Whole body and SPECT/CT images were acquired 24 and 48 h after injection of ^111^In-labeled IMP288 **(A,C)** and ^177^Lu-labeled IMP288 **(B,D)**. Patient 1 included in the first cohort had SCLC and a CEA level of 79 ng/mL. These images showed low tumor targeting in liver, mediastinum, and lung metastases.

**Figure 4:**
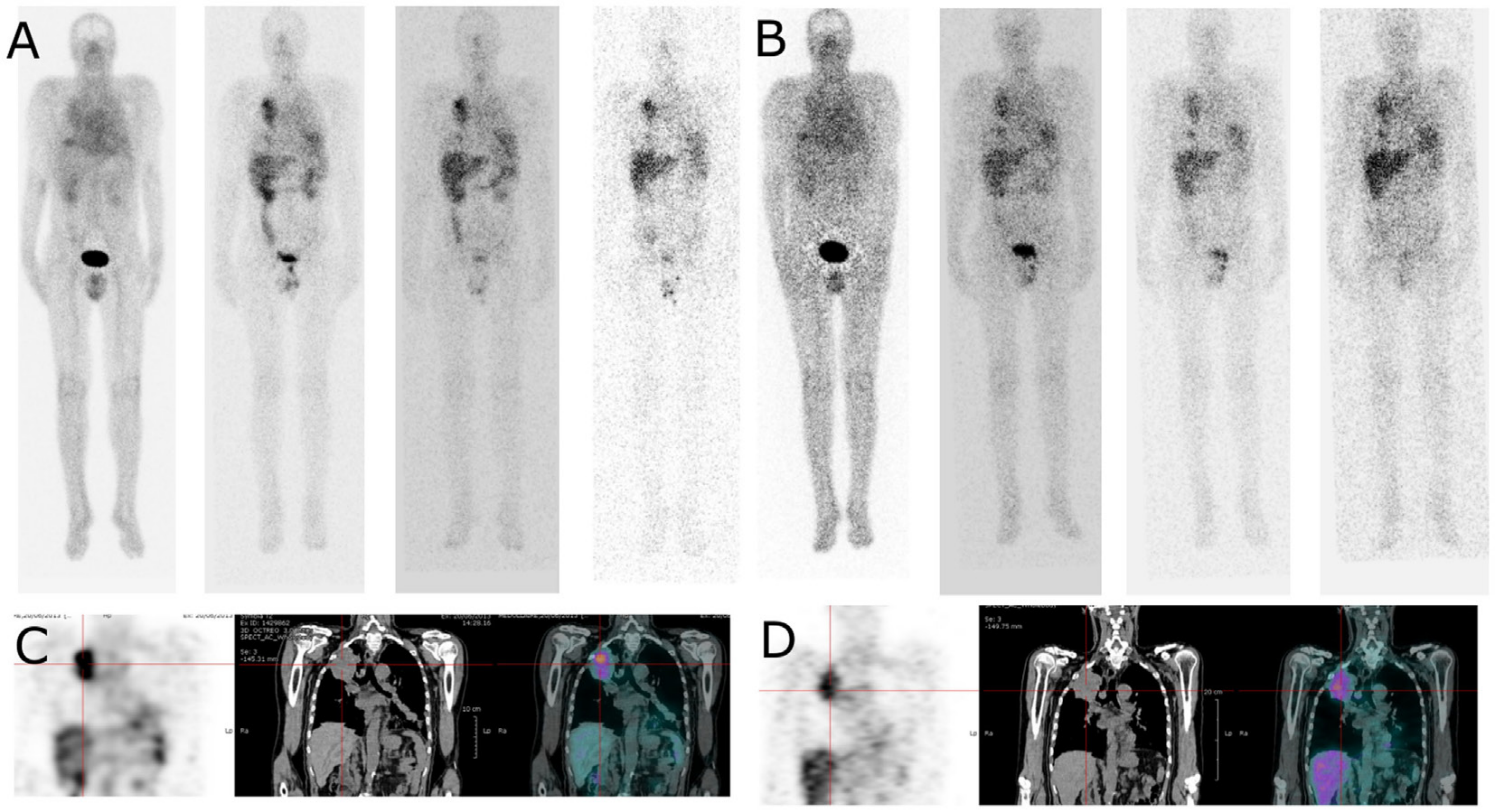
**Whole body and SPECT/CT images of patient 4 (cohort II)**. Whole body images (anterior view) were acquired 4, 24, 48, and 72 h after injection of ^111^In-labelled IMP288 **(A)** and ^177^Lu-labelled IMP288 **(B)**. Patient 4, included in the cohort II, had NSCL and a CEA level of 275 ng/mL. These images clearly shows lung tumor targeting in whole body and SPECT/CT images [**(C)** with ^111^In-labelled IMP288 and **(D)** with ^177^Lu-labelled IMP288]. According to RECIST criteria, the disease was considered as stable at 4 weeks, but progressive at 3 months. Patient 4 was the only patient with HAHA against TF2 > 50 ng/mL detected 1 month after the last TF2 injection.

Images registered after ^111^In-labeled IMP288 showed targeting of all known tumors in all patients. WB scintigraphy and SPECT/CT images recorded after S1 and S2 were concordant (example of patients included in C1 and C2 are shown in Figures 3 and 4) even if tumor targeting visually appeared to be better in S2 than in S1 images, due to the higher levels of administered activity.

Reconstructed tomographic images were quantified and indium-111 counts were also corrected for radioactive decay to allow for comparison with the lutetium-177 data. A two-compartment model was used to describe the kinetics of WB counts (Table 6). The mean alpha half-life was 4.3 ± 1.1 h and beta half-life 80 ± 7 h, corresponding to a relatively large interindividual variability (25 and 9%, respectively).

**Table 6:** **Two-compartment population analysis of whole body IMP288 activity pharmacokinetics**.

Parameter^a^	*k*_2,1_ (h^−1^)	*k*_1,2_ (h^−1^)	*A*_WB_	*B*_WB_	MR	*k*_el_ (h^−1^)
**Population values**						
Estimation	0.0163	0.0096	0.105	0.14	NA	NA
SD	0.0014	0.0009	0.004	0.01	NA	NA
**Individual values**						
Patient 1, indium	0.0152	0.0101	0.097	0.13	124.2	0.18
Patient 1, lutetium	0.0177	0.0094	0.110	0.15	125.5	0.23
Patient 2, indium	0.0126	0.0100	0.104	0.14	40.7	0.18
Patient 2, lutetium	0.0138	0.0101	0.115	0.16	40.1	0.21
Patient 3, indium	0.0178	0.0090	0.115	0.16	35.7	0.20
Patient 3, lutetium	0.0197	0.0085	0.121	0.17	37.8	0.23
Patient 4, indium	0.0136	0.0099	0.097	0.14	22.6	0.15
Patient 4, lutetium	0.0166	0.0099	0.089	0.13	22.0	0.13
Patient 5, indium	0.0146	0.0104	0.098	0.14	22.5	0.15
Patient 5, lutetium	0.0165	0.0102	0.083	0.12	31.1	0.13
Patient 6, indium	0.0141	0.0101	0.071	0.11	109.0	0.12
Patient 6, lutetium	0.0166	0.0098	0.091	0.13	18.8	0.13
Patient 7, indium	0.0143	0.0101	0.102	0.14	3.3	0.12
Patient 7, lutetium	0.0191	0.0090	0.070	0.13	3.2	0.08
Patient 8, indium	0.0141	0.0100	0.110	0.15	2.3	0.12
Patient 8, lutetium	0.0190	0.0090	0.098	0.14	2.5	0.11
Mean^b^	0.0160	0.0097	0.098	0.14		0.15
SD	0.0022	0.0006	0.015	0.02		0.04
CV (%)	13.9	5.8	15.2	11.6		29.0

*^a^Whole body (WB) activity kinetics was modeled as the sum of the activities in a central compartment and a distribution compartment with transfer rates (*k*_2,1_ and *k*_1,2_), and two parameters *A* and *B* as adjustable parameters; the elimination constant *k*_el_ was calculated as A_S_ × MR^BS^, MR being the ratio of the number of moles of injected IMP288 to the number of moles of TF2 present in the circulation at the time of IMP288 injection. Estimations of the population adjusted parameters are given together with their estimated SD*.

*^b^The mean, SD, and CV for each parameter were calculated from individual estimations*.

The activity in the organs was modeled by a fraction of the activity in the central compartment plus an organ-specific distribution compartment, as described in the literature (27). Then the kinetics of activities in all regions of interest (WB, right and left lungs, liver, right and left kidneys, spleen, heart, whole aorta, and tumor), for all eight patients and for the two sessions, was fitted simultaneously by a population analysis. As in the serum kinetics analysis, the rate constant of elimination (*k*_el_) from the central compartment was set as a power function of MR, and tissue weights were introduced in the analysis. A reasonable fit was obtained, except for tumors, for which variations in on rates and off rates were too large to consider a population PK analysis and were thus adjusted individually. This analysis demonstrated the consistence of the measured activities and a relationship between WB clearance, and MR was again observed [*k*_el_ (1/h) = 0.095 MR^0.15^], but with a lower correlation coefficient (*R*^2^ = 0.45) than in the serum count analysis.

This analysis showed that tissue uptake, on an organ weight basis, as assessed by the organ compartment volume, was higher in kidneys and liver, as expected, intermediate in lungs and spleen, and lower in aorta and heart, whereas the fractions of activity in fast equilibrium with the central compartment was similar in all tissues, including tumors (Table 7). Tumor uptake was variable and higher in patients 4, 5, 6 (C1), and 7 (C2).

**Table 7:** **Population analysis of IMP288 activity distribution**.

Parameter^a^	*k*_on_ (1/h × 10^3^)	*k*_off_ (1/h × 10^2^)	Fraction (L/kg × 10^2^)
Lung	1.06 ± 0.06	1.17 ± 0.06	3.38 ± 0.19
Liver	1.45 ± 0.11	1.14 ± 0.08	2.33 ± 0.21
Kidneys	4.35 ± 0.28	2.22 ± 0.08	5.91 ± 0.37
Spleen	1.48 ± 0.11	1.30 ± 0.08	2.21 ± 0.19
Heart	0.45 ± 0.04	0.94 ± 0.09	3.08 ± 0.21
Aorta	0.61 ± 0.05	1.04 ± 0.09	3.66 ± 0.26
Tumor			2.70 ± 0.26

*^a^IMP288 distribution in tissues of interest was modeled using a tissue-specific distribution compartment and a fraction of the activity in the central compartment; the on and off rate constants, *k*_on_ and *k*_off_, and the fraction of activity (fraction) were adjustable parameters using the central compartment of the whole body kinetics as the input function*.

### Dosimetry

**Figure 5:**
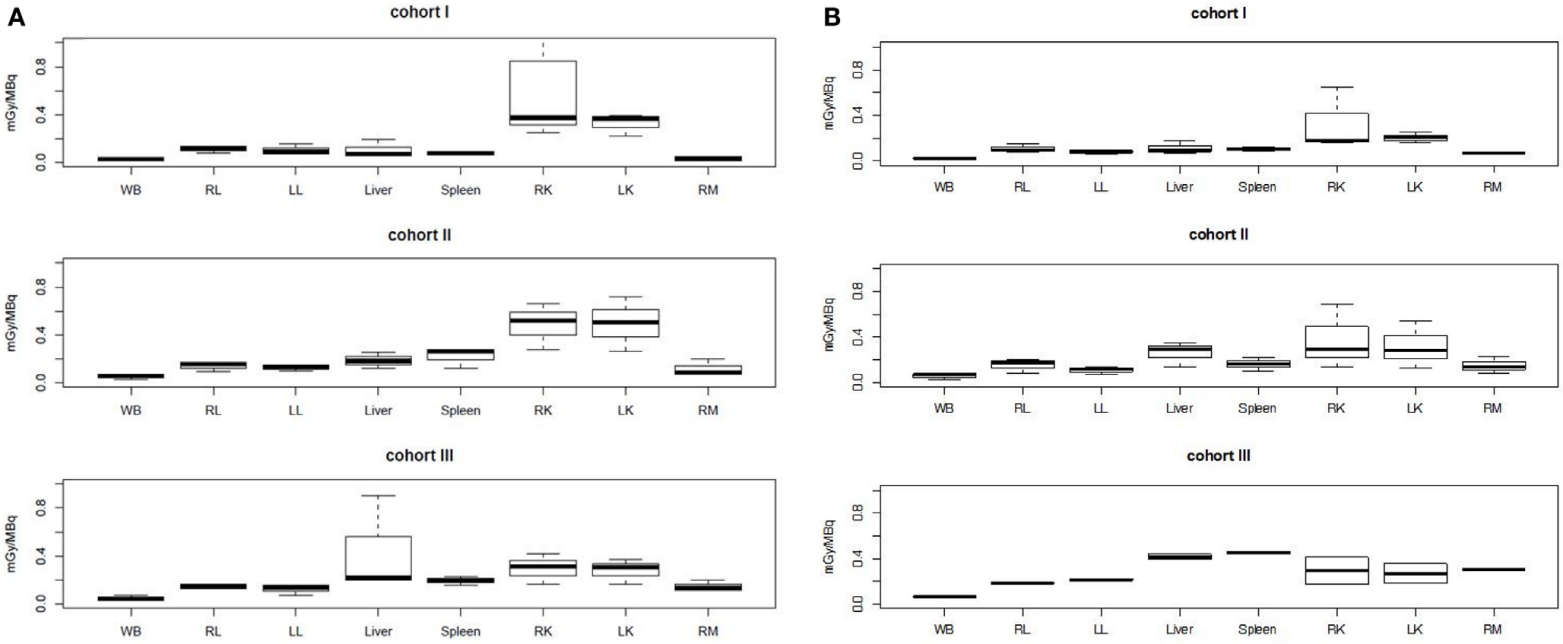
**Normalized organ-absorbed doses per cohort assessed in the pretherapeutic (A) and therapeutic sessions (B)**. No significant statistical differences were found between groups. Abbreviations: WB, RL, LL, RK, LK, and RM stand, respectively, for whole body, right lung, left lung, right kidney, left kidney, and red marrow.

**Figure 6:**
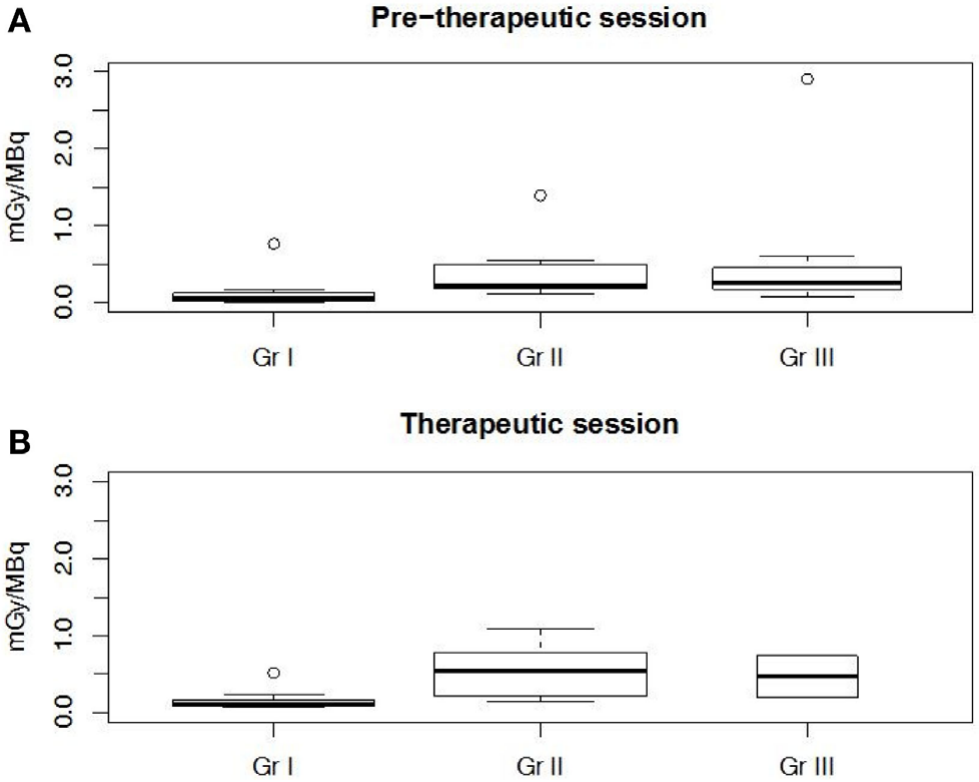
**Tumor normalized-absorbed doses per cohort assessed in the pretherapeutic (A) and therapeutic sessions (B)**. Cohort I was significantly (*p* < 0.002) different from cohorts II and III.

The PK modeling of image data was completed by a classical dosimetry study performed as described in Section “Materials and Methods.” Figures 5 and 6 present organ-absorbed doses normalized by injected activity estimated at S1 and S2. Considering cohort categorization, no significant differences in organ-absorbed doses estimated from both sessions were observed, as shown by Wilcoxon tests (*p* > 0.05). At the patient level, normalized organ-absorbed doses at S1 were also compared against those estimated at S2, using the Spearman test. Spearman’s rho and corresponding *p*-value are reported in Table 8 and showed a good correlation between organs-absorbed doses estimated at both sessions.

**Table 8:** **Spearman tests for the correlation between organ-absorbed doses estimated during pretherapeutic and therapeutic sessions^a^**.

Spearman test	1	2	3	4	5	6	7	8
Rho	0.986	0.827	0.904	0.725	0.918	0.836	0.891	0.727
*p*-Value	0.000	0.031	0.000	0.007	0.000	0.003	0.001	0.015

*^a^Patient 9 was not able to sustain therapeutic imaging session due to an altered condition*.

For all patients, 24 tumors were delineated on reconstructed SPECT or CT images. They were mostly located in lungs (37%) and liver (29%). Median tumor mass was 19.3 g (1.1–1,364 g). Median normalized tumor-absorbed doses in the three cohorts ranged from 0.05 to 0.25 mGy/MBq in S1 and from 0.10 to 0.54 mGy/MBq in S2. Extreme normalized tumor-absorbed doses for each cohort ranged from 0.02 to 0.12 mGy/MBq in S1 and from 0.52 to 1.08 mGy/MBq in S2. These absorbed doses not appeared to be significantly different as shown by a Wilcoxon test conducted on previous values (*p* > 0.13). Nevertheless, a Spearman test conducted on previous data showed a good correlation (rho = 0.85, *p* < 10^−15^) between tumor-absorbed doses estimated at both sessions.

Kruskal–Wallis tests were performed to compare organ-absorbed doses at each different dosage level and for both sessions. Statistical tests did not exhibit significant difference between groups for organs (*p* > 0.07). The same analysis was conducted on tumor-absorbed doses and showed significantly higher tumor-absorbed doses in patients included in cohort II or III than in cohort I (*p* < 0.002) as illustrated by Figure 6.

## Discussion

The main objectives of this study were to assess whether an imaging session may be predictive of absorbed doses in therapy (IMP288 labeled with indium-111 in the imaging session and with lutetium-177 in the therapy session) and to find the best pretargeting parameters (molar doses of TF2 and IMP288 and pretargeting delay) for cancer therapy. The imaging sessions were performed using molar doses 5.4 times lower than in the therapy session, to reduce exposure of the patients to the pretargeting reagents. This was assumed feasible without compromising the predictive character of the imaging session, based on the earlier preclinical assessment of the compounds and in view of the results obtained by Schoffelen et al. (16). It was, however, important to confirm this result in a different clinical setting.

To this end, PK modeling of TF2 serum concentration kinetics, IMP288 serum activity kinetics (adjusted to the physical half-life of lutetium-177 to allow comparisons), and of IMP288 activities measured by SPECT in WB and regions of interest corresponding to major tissues and tumors was performed using a population PK modeling software developed in our laboratory (available upon request) that allowed for simultaneous multicompartment and multitissue analyses. The population approach allowed a more consistent modeling of data, ensuring convergence of parameter estimations even in cases where the number of data points was small due to assay sensitivity (TF2) or number of imaging sessions. In addition, the population approach allowed us to confirm that calculating individual TF2 or hapten doses on the basis of BSA reduced the variability of circulating TF2 concentrations and also allowed us to define a relationship between circulating TF2 concentrations at the time of hapten administration and the kinetics of the hapten. This relationship is complex, but it is expected that TF2 binds the hapten in the circulation and slows down its clearance, as already observed in many preclinical studies and in earlier clinical studies with chemically coupled bispecific antibodies (17).

The population PK analyses showed that molar ratios and time intervals must be kept constant, but that there is no need to perform the imaging and therapy sessions with the same molar doses, in order to obtain comparable hapten serum and tissue uptake kinetics in both imaging and therapy sessions. Indeed, quantitative assessment of the images showed that not only the serum kinetics, but also the distribution of activity in normal tissues and tumors in the therapy session may be predicted from the imaging session, as shown by the very high degree of correlation obtained. Being able to predict dosimetry, and avoiding treatment of patients whose tumor lesions do not bind the activity, using a low dose, pretherapy, imaging session, according to the theranostic approach, is a significant advantage in clinical practice.

The very different PK behavior of TF2 as compared to chemically coupled BsmAb requires pretargeting optimization studies. The pretargeting parameters must be finely tuned to avoid high circulating activities, detrimental to normal tissue dosimetry in therapy, and background in imaging, while keeping tumor uptake as high as possible. In this study, in spite of the small number of patients in each cohort, some conclusions can be drawn. In cohort I, comparatively low doses of TF2 were administered (7 and 37.5 mg/m^2^) with a delay of 48 h. This results in a fast clearance of the hapten and a low tumor uptake. In cohort II, the higher doses of TF2 (14 and 75 mg/m^2^) resulted in slower clearance of activity and significantly increased tumor uptake. Reduction of the delay to 24 h further increased the amount of TF2 present in the circulation and tissues at the time of hapten injection but did not significantly increased tumor uptake over cohort III, probably because of the high observed variability and because lutetium-177 data were available for only two patients.

Although serum and organ PK showed a correlation between MR and clearance, it must be noted that between cohort I and cohort III, clearance is only reduced by a factor of approximately two. In contrast, a significant difference in tumor-absorbed doses is found between cohort I and cohorts II and III, showing that increase of the TF2 dose and reduction of the pretargeting delay had a positive impact on tumor uptake without increasing significantly the doses delivered to normal tissues.

In line with the dosimetry assessment, hematological toxicity was quite limited, and non-hematological toxicity was observed only in liver involved with tumor in three cases. To further increase tumor-absorbed doses, the shorter pretargeting delay of 24 h thus appears superior to 48 h. Since in preclinical models (28), shorter delays started to increase the doses delivered to normal tissues, this 24-h delay may be considered optimal. On the other hand, increasing the administered amount of TF2 could be problematic in terms of cost, even if there is no indication of tumor binding site saturation. Thus, to improve treatment efficacy, which was minimal in this optimization study with only two cases of disease stabilization for short periods of time, the injected activity should be increased for the second part of the study, which is planned with an activity escalation. Indeed, it was not expected that a single therapy cycle would be sufficient to deliver therapeutic doses that would show tumor shrinkage.

It has been shown that pretargeting performance strongly depends on the injected hapten to BsmAb molar ratio which should be kept as small as possible (1:20 or 1:40), as in this study. Then the hapten should be labeled to a high specific activity in order to target enough activity for therapy. This specific activity issue and the fact that tumor accretion is slower but significantly reversible make the use of shorter half-life and higher intrinsic toxicity radionuclides, such as yttrium-90, preferable to that of lutetium-177. The use of pairs of beta + /beta-emitting radionuclides (e.g., ^86^Y/^90^Y) could also be a promising theragnostic approach with a same distribution both for dosimetry imaging using immunoPET and therapy. Short half-life alpha-emitting radionuclides, such as bismuth-213 or astatine-211, could also be considered.

Finally, the immune response and the number of patient developing human antibody against TF2 were lower than described previously by Schoffelen and coworkers (16) in a population of patients with CRCs, and grade 2 acute infusion reactions were observed in one-third (7/21) of the patients during the second TF2 infusion. These immune reactions were attenuated by adding antihistamine and corticosteroid premedications before the second TF2 infusion but did not disappear (immune response occurred in 2/5 premedicated patients). In our study, all patients were premedicated with an antihistamine and corticosteroid before each TF2 and peptide infusion, and none of them presented immune response symptoms. Moreover, Schoffelen and coworkers (16) described an immunization rate of 50% with HAHA against TF2 detected in 11/21 patients as soon as 1 week after the second infusion gradually increasing in the follow-up period of 8 weeks. HAHA against TF2 > 50 ng/mL was detected in only one of our eight patients evaluated, starting 1 month after the second TF2 infusion and gradually decreasing in the follow-up period of 3 months. If the use of an antiallergic premedication partially explained the absence of immune reactions at the time of second injection of TF2 in our study, this did not explain the lower number of patients developing HAHA. The explanation did not come from the TF2 injection schemes which were identical or from the TF2 doses that were close. Our main hypothesis to explain this discrepancy could reside in the different histological types of tumors included in the two studies. While in the two studies, patients have already been treated with multiple lines of treatment, relapsing lung cancer is often more aggressive than relapsing CRC. This could explain poorer general conditions (7/9 patients died in the year following the study) and thus a more compromised immune system. It may also be that metastatic NSCLC patients are more heavily pretreated with cytotoxic chemotherapy than patients with metastatic CRC, and this needs to be evaluated. In any case, the low immunogenicity and the low toxicity observed should offer the possibility of administering several courses of treatment to deliver tumor-killing irradiation levels.

## Conflict of Interest Statement

The proprietary TF2 and IMP288 reagents were provided by Immunomedics, Inc., and IBC Pharmaceuticals, Inc. (Dr. David M. Goldenberg and Robert M. Sharkey).

## Abbreviations

ALT, alanine transaminase; AST, aspartate transaminase; BSA, body surface area; BsmAb, bispecific monoclonal antibody(ies); CEA, carcinoembryonic antigen; Cl, clearance; CRC, colorectal cancer; CT, computed tomography; DOTA, 1,4,7,10-tetraazacyclododecane-1,4,7,10-tetraacetic acid; DTPA, diethylene-triamine-pentaacetic acid; EGFR, epidermal growth factor receptor; FDG-PET, positron emission computed tomography using ^18^F-fluoro-deoxy-glucose; HAHA, human antihuman antibody; HAMA, human antimouse antibody; HSG, histamine-succinyl-glycine; mAb, monoclonal antibody(ies); MR, molar ratio of injected hapten to the amount of TF2 present in the patients’ serum; MTC, medullary thyroid carcinoma; NSCLC, non-small-cell lung cancer; PET, positron emission computed tomography; PK, pharmacokinetic(s); pRAIT, pretargeted radioimmunotherapy; RAIT, radioimmunotherapy; SCLC, small-cell lung cancer; SD, standard deviation; SPECT, single photon emission computed tomography; TF2, anti-CEA × anti-HSG bispecific monoclonal antibody; Vc, central compartment volume; WB, whole body.
