# Supplementary material for: Pharmacokinetics and Dosimetry Studies for Optimization of Pretargeted Radioimmunotherapy in CEA-Expressing Advanced Lung Cancer Patients
Source: Front Med (Lausanne). 2015 Nov 27;2:84. doi: 10.3389/fmed.2015.00084 (PMC4661432; doi:10.3389/fmed.2015.00084)
Supplement: Supplementary file 1 [file Presentation_1.PDF]

SUPPLEMENTARY MATERIAL

TABLES

Table 1: Median and extreme organ absorbed doses normalized by injected activity estimated from the pre-therapeutic session.

mGy/mBq	WB	Right lung	Left lung	Liver	Spleen	Right kidney	Left kidney	Red marrow
Cohort I N=3	0.03 [0.02, 0.03]	0.12 [0.07, 0.13]	0.09 [0.07, 0.15]	0.07 [0.06, 019]	0.08 [0.06, 0.08]	0.37 [0.25, 1.32]	0.37 [0.22, 0.33]	0.08 [0.01, 0.03]
Cohort II N=3	0.05 [0.02, 0.07]	0.15 [0.09, 0.16]	0.13 [0.10, 0.14]	0.18 [0.12, 0.26]	0.26 [0.12, 0.26]	0.52 [0.27, 0.66]	0.51 [0.26, 0.72]	0.08 [0.08, 0.19]
Cohort III N=3	0.04 [0.03, 0.07]	0.14 [0.14, 0.15]	0.14 [0.07, 0.15]	0.22 [0.20, 0.90]	0.20 [0.15, 0.22]	0.31 [0.16, 0.42]	0.30 [0.16, 0.37]	0.13 [0.12, 0.20]

Table 2: Median and extreme organ organ absorbed doses (mGy/MBq) estimated from the therapeutic session[a].

mGy/mBq	WB	Right lung[b]	Left lung	Liver	Spleen	Right kidney	Left kidney	Red marrow
Cohort I N=3	0.02 [0.02, 0.03]	0.09 [0.08, 0.15]	0.08 [0.06, 0.09]	0.09 [0.07, 018]	0.10 [0.09, 0.12]	0.18 [0.16, 0.65]	0.20 [0.16, 0.25]	0.07 [0.06, 0.07]
Cohort II N=3	0.07 [0.03, 0.07]	0.17 [0.08, 0.20]	0.11 [0.07, 0.14]	0.29 [0.14, 0.35]	0.16 [0.10, 0.22]	0.30 [0.14, 0.69]	0.28 [0.13, 0.54]	0.14 [0.09, 0.23]
Cohort III N=2	0.07 [0.06, 0.07]	0.18 [0.18, 0.18]	0.22 [0.21, 0.23]	0.42 [0.39, 0.44]	0.45 [0.44, 0.46]	0.29 [0.18, 0.41]	0.27 [0.18, 0.36]	0.31 [0.31, 0.31]

a: Patient 9 was not able to sustain therapeutic imaging session due to an altered condition, b: Patient 8 had no right lung.

FIGURES

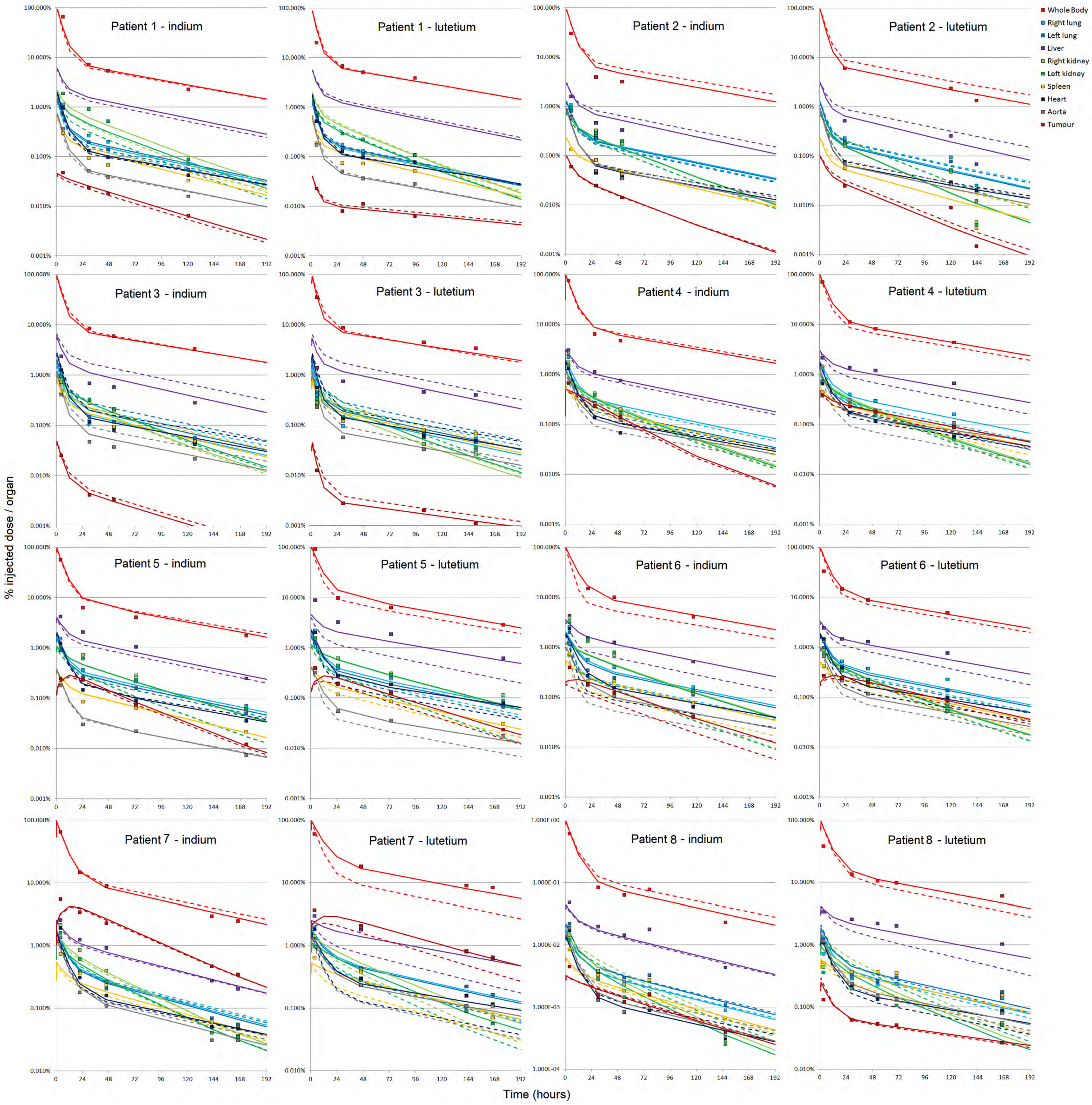

F

Figure 1: Biodistribution kinetics from image quantification. Each patient received TF2 infusions then 24 or 48 hours after each infusion, they received an infusion of IMP288 labelled with indium-111 after the first TF2 infusion or labelled with lutetium-177 after the second. Tomograhic images were recorded at selected time intervals, segmented and quantified. Activity kinetics in organs of interest and tumours were modelled using a two-compartment for Whole Body, and each organ was modelled as an un uptake compartment plus a fraction of the central compartment activity using a population approach. Tumour uptake parameters were fitted individually. Data are shown as coloured squares as a semi-log plot together with the population (dashed lines) and individual (solid lines) fitted curves.

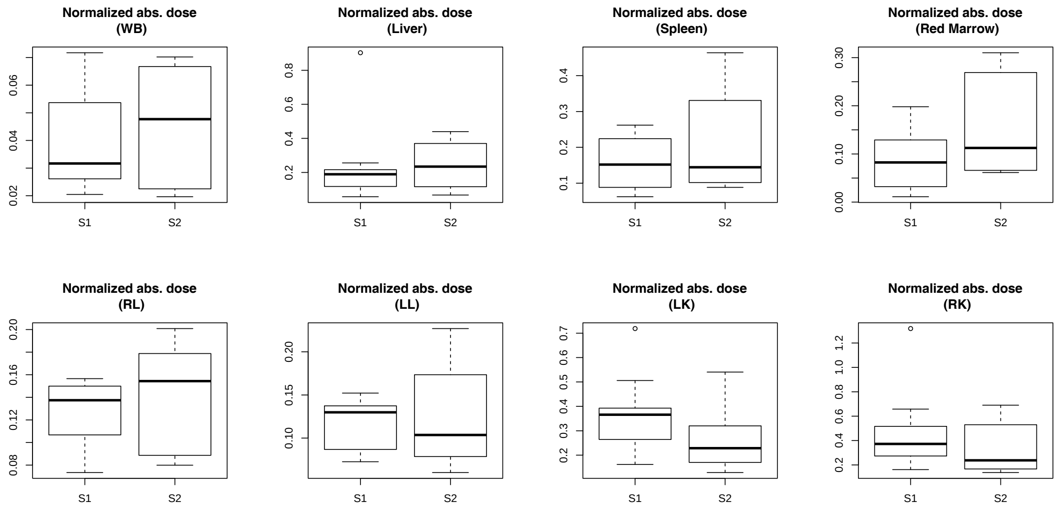

Figure 2: Normalized organ absorbed doses estimated during pre-therapeutic and therapeutic session. Abbreviations: WB, RL, LL, RK and LK stand respectively for Whole-Body, right lung, left lung, right kidney, left kidney.
